# Post-COVID-19 Yearly Pattern Changes and Gender Variations in Attention Deficit Hyperactivity Disorder Patients at an Urban Mental Health Clinic in Alabama, USA

**DOI:** 10.7759/cureus.69596

**Published:** 2024-09-17

**Authors:** Rasheeq M Amin, Sharno C Amin, Nasima J Amin, M. Aminul Islam

**Affiliations:** 1 Psychiatry and Behavioral Sciences, Alabama College of Osteopathic Medicine, Birmingham, USA; 2 Psychiatry and Behavioral Sciences, My Psychiatry Clinic, Hoover, USA

**Keywords:** psychiatry & mental health, gender comparison, non-stimulant, stimulant treatment, attention deficit hyperactivity disorder (adhd)

## Abstract

Introduction

The COVID-19 era has seen an increased trend in attention deficit hyperactivity disorder (ADHD) diagnoses. Historically, males have been diagnosed with ADHD more frequently than females during childhood. Studies have indicated a higher use of stimulant medications among male ADHD cases compared to females. This study examines ADHD cases from 2021 to 2023 to analyze yearly trends following the initial COVID-19 spike and explores gender and age differences between ADHD-positive and ADHD-negative cases.

Methods

This retrospective study was conducted using data from an urban outpatient mental health clinic in Alabama. Data were extracted from Electronic Health Records (EHR) for patients seen from January 1, 2021, to December 31, 2023. The Institutional Review Board (IRB) approved the study under the exempt research category. Data were analyzed using Microsoft Excel (Microsoft® Corp., Redmond, WA, USA) and the Statistical Package for the Social Sciences (IBM SPSS Statistics for Windows, IBM Corp., Version 26.0, Armonk, NY). Diagnoses were based on the Diagnostic and Statistical Manual of Mental Disorders, Fifth Edition (DSM-5) criteria, and clinical diagnoses and medication information were obtained from the EHR.

Results

The study included 1,422 patients, of whom 881 (62%) were diagnosed with ADHD. Females with ADHD had significantly higher comorbid conditions, such as major depressive disorder, generalized anxiety disorder, panic disorder, and post-traumatic stress disorder, compared to males with ADHD. Gender differences in ADHD diagnoses were seen over the years, though no significant age differences were observed.

Conclusions

The study indicates a sustained high rate of ADHD diagnoses even after the initial COVID-19 spike. Females showed a higher ADHD diagnoses compared to males, but stimulant medication use remained consistent across genders. No significant age differences were observed between males and females with ADHD. Further research is needed to explore the reasons behind these gender differences and to evaluate their implications.

## Introduction

Attention deficit hyperactivity disorder (ADHD) is characterized by persistent symptoms of inattention and/or hyperactivity and impulsivity that impair daily functioning and quality of life [1,2]. ADHD is typically diagnosed in childhood, with approximately 9.4% of children and adolescents in the United States affected [3]. While young children with ADHD primarily exhibit hyperactivity and impulsivity, inattention becomes more prominent with age [4]. For a diagnosis of adult ADHD, symptoms must have been present before age 12, although they may not have been recognized during childhood. Adult ADHD symptoms can be less overt, with hyperactivity often diminishing but challenges with impulsiveness, restlessness, and inattentiveness persisting. This condition can lead to difficulties in relationships, work or academic performance, low self-esteem, and reduced productivity.

Previous studies have documented an increase in ADHD symptoms during the COVID-19 pandemic [5], attributed to heightened stress, social isolation, and altered home and work environments. It was anticipated that symptoms would persist beyond the initial pandemic surge [6]. However, few studies have examined ADHD prevalence beyond 2020. This study investigates ADHD diagnosis trends from 2021 to 2023.

ADHD is more commonly diagnosed in males, but recent trends indicate an increase in diagnoses among females, particularly of the inattentive type. ADHD patients frequently have comorbid conditions such as learning disabilities, anxiety disorders, conduct disorders, depression, and substance use disorders [7]. This study explores gender differences and stimulant medication use patterns over the three years following the COVID-19 pandemic spike in 2020.

## Materials and methods

Data were obtained from the Electronic Health Records (EHR) system of an urban outpatient psychiatry clinic in Birmingham, Alabama, USA. The clinic, located in the heart of the city, provides mental health services for all patients who seek it. Patients were evaluated by board-certified psychiatrists using the Diagnostic and Statistical Manual of Mental Disorders, Fifth Edition (DSM-5) criteria and were treated with psychotropic medications based on their practicing experience. Data from all patients seen between January 1, 2021, and December 31, 2023, were entered in a Microsoft Excel spreadsheet (Microsoft® Corp., Redmond, WA, USA). This time frame was selected to capture patient trends post-initial COVID-19 spike in 2020. The study used first-visit data; no follow-up information was included, and no subjects were excluded.

Variables included age, gender, and insurance status. The age range was 2 to 29, with 3.2% categorized as children (under 19 years). Age was categorized using a cutoff value of 35, which was the mean age. Gender was categorized as male and female. Insurance status served as a proxy for socio-economic status with patients without insurance categorized as having no insurance, even if they paid out-of-pocket. ADHD included all patients diagnosed with ADHD, predominantly inattentive type (International Classification of Diseases, 10th Revision (ICD 10) code F90.0); ADHD, predominantly hyperactive type (ICD 10 code F90.1); and ADHD, predominantly combined type (ICD 10 code F90.2). Major depressive disorders (MDD) included all patients diagnosed with MDD, recurrent, mild (ICD 10 code F33.0); MDD, recurrent, moderate (ICD 10 code F33.1); MDD, recurrent, severe without psychotic features (ICD 10 code F33.2); and MDD, recurrent, severe with psychotic features (ICD 10 code F33.3). Anxiety disorders were separately categorized as generalized anxiety disorder (GAD) (ICD 10 code F41.1), panic disorder (Panic D/O) (ICD code F41.0), and post-traumatic stress disorder (PTSD) (ICD 10 codes, F43.10, F43.11, and F43.12). Opioid use disorders included opioid abuse, uncomplicated (ICD 10 code F11.10); and opioid dependence, uncomplicated (ICD code 10 F11.20). Similarly, alcohol use disorders (ETOH) included alcohol abuse, uncomplicated (ICD 10 code F10.10;) and alcohol dependence, uncomplicated (ICD code 10 F10.20). All other substance use disorders (other SUD) predominantly included stimulant abuse, uncomplicated (ICD 10 code F15.10); stimulant dependence, uncomplicated (ICD 10 code F15.20); cannabis abuse, uncomplicated (ICD 10 code F12.10); cannabis dependence, uncomplicated (ICD 10 code F12.20); sedative, hypnotic or anxiolytic abuse, uncomplicated (ICD 10 code F13.10); sedative, hypnotic or anxiolytic dependence, uncomplicated (ICD 10 code F13.20); cocaine abuse, uncomplicated (ICD 10 code F14.10); and cocaine dependence, uncomplicated (ICD 10 code F14.20). Stimulant medications used for treating ADHD included methylphenidate, dextroamphetamine-amphetamine, and lisdexamfetamine, among others. Non-stimulant medications used for treating ADHD-positive cases included atomoxetine, guanfacine, bupropion, clonidine, and others.

Data analysis was performed using the Statistical Package for the Social Sciences (IBM SPSS Statistics for Windows, IBM Corp., Version 26.0, Armonk, NY). Qualitative data were expressed as numbers and percentages, and Pearson's Chi-squared test was used for statistical significance. Quantitative data were expressed as mean and standard deviation (mean ± SD), with differences measured using t-tests. A p-value of less than 0.05 was considered statistically significant. Ethical approval was obtained from the WIRB and Copernicus Group IRB, as an exempt study (approval #1-1789150-1; date of approval: August 1, 2024).

## Results

The study analyzed data from 1,422 patients seen between 2021 and 2023, of whom 881 (62%) were diagnosed with ADHD. Table 1 details the characteristics of ADHD-positive and negative cases by gender.

**Table 1:** Characteristics of ADHD-positive and ADHD-negative patients by gender (values are expressed as percentages). Significance: *P<0.05, **P<0.01, ***P<0.001 by Pearson's Chi-square test; ADHD: attention deficit hyperactivity disorder; MDD: major depressive disorder; GAD: generalized anxiety disorder; PTSD: post-traumatic stress disorder; ETOH: alcohol use disorder; SUD: substance use disorder; D/O: disorder

Parameters	ADHD positive		ADHD negative		Total patients	
	(N=881)		(N=541)		(N=1422)	
Gender	Male	Female	Male	Female	Male	Female
Age ≤35 in years	54.2	59.0*	49.1	50.8	52.2	55.9
Age >35 in years	45.8	41	50.9	49.2	47.8	44.1
Insured	77	81.7	77.6	83.8	77.2	82.5
MDD	58.2	65.7***	73.4	79.5	64.2	70.8
GAD	42.4	52.5**	54.2	62.7	47.1	56.3
Panic D/O	21.8	24.0***	36.9	42.2	27.8	30.8
PTSD	11.8	28.5**	16.8	39.1	13.8	32.5
ETOH	3.0*	1.8	12.6	4.3	6.8	2.7
Opioid Use	6.7	3.1	11.7	2.4	8.6	2.8
Other SUD	1.5	1.8	8.4**	0.2	4.2	0.8
Bupropion	27.6	26	27.1	30	27.4	27.4

**Figure 1:**
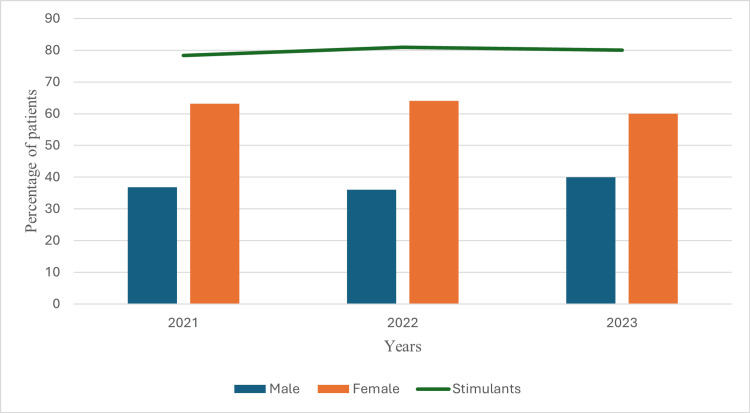
Yearly pattern of percentages of ADHD-positive cases among males and females by stimulant medications prescribed. N=881, differences were significant at P<0.05 by Pearson's Chi-square tests; ADHD: attention deficit hyperactivity disorder

ADHD-positive females had significantly higher comorbid conditions, including MDD, GAD, panic disorders, and PTSD, compared to ADHD-positive males. No such differences were observed among ADHD-negative patients. Females showed a higher representation among ADHD patients over the years (Figure 1).

**Figure 2:**
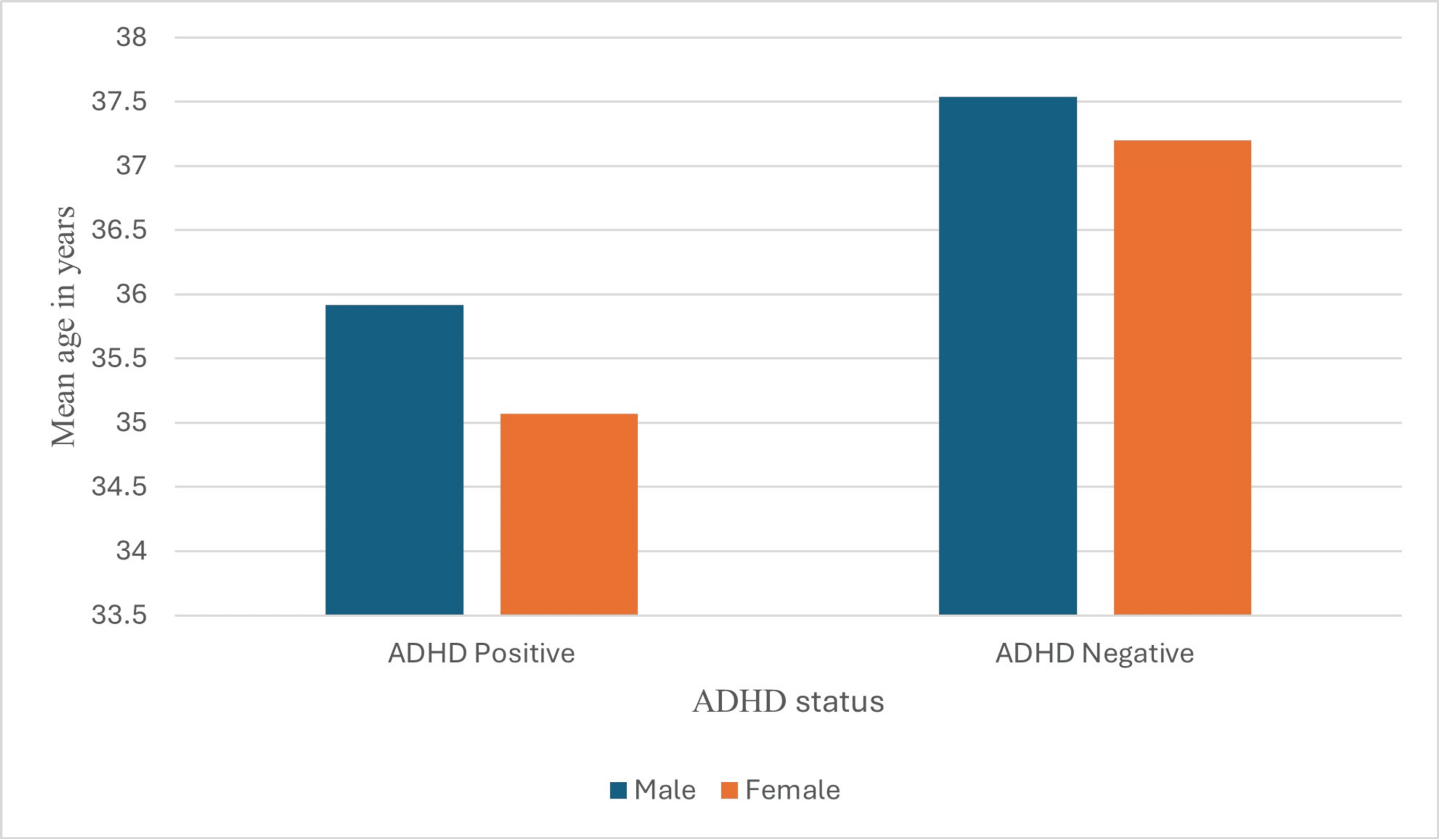
Mean age differences of both ADHD-positive and ADHD-negative patients among males and females. N=1422, P>0.05 by independent t-test; ADHD: attention deficit hyperactivity disorder

The line graph in Figure 1 illustrates the consistent prescription pattern of stimulant medications (approximately 80%) from 2021 to 2023, regardless of gender. The proportion of ADHD-positive cases remained stable during this period (57.2%, 65.7%, and 65.3% in 2021, 2022, and 2023, respectively). Although females under 35 were more frequently diagnosed with ADHD, the mean age of ADHD-positive and negative cases was similar across genders (Figure 2).

**Figure 3:**
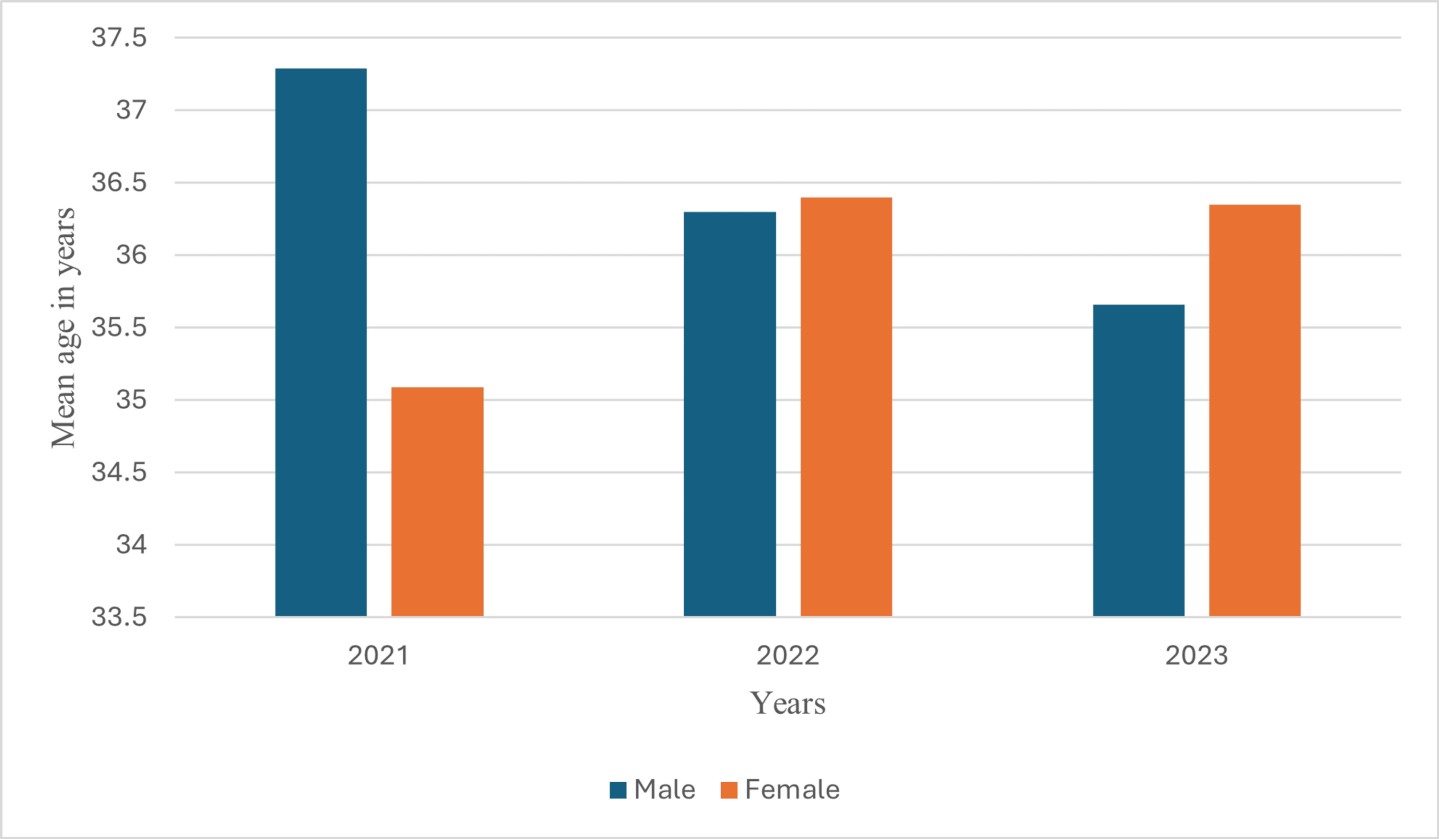
Yearly pattern of mean age differences among ADHD-positive patients by gender. N=881, P>0.05 by independent t-test; ADHD: attention deficit hyperactivity disorder

Furthermore, there were no significant age differences between genders over the years (Figure 3).

## Discussion

ADHD in adults

ADHD is marked by difficulties with inattention, with or without hyperactivity and impulsivity [1]. Despite symptoms often starting in childhood, diagnosis during this period may be hindered by a lack of awareness or stigma associated with mental illness. As such a significant number of children continue to exhibit the symptoms into adulthood [8]. Diagnostic criteria for ADHD in adults remain similar to those used for children, requiring at least five symptoms of inattention, hyperactivity, and impulsivity [9]. Diagnostic procedures for adults with ADHD use standard rating measures to assess symptoms occurring within the last six months. These symptoms must impact the individual’s ability to perform at work or school, affect relationships with family and friends, and influence personal life and well-being [10]. ADHD in adults can also be obscured by co-occurring mental health issues. Depression, anxiety disorder, bipolar disorder, and personality disorders are prevalent among adults with ADHD [11]. Additionally, the high prevalence of SUD among adults complicates the diagnosis of ADHD. SUD involves physical dependence on substances such as alcohol, cannabis, cocaine, opioids, amphetamines, barbiturates, and hallucinogens. The dual diagnosis of mental illness and SUD has increased among adults over the past decade [12]. To confirm the diagnosis of ADHD, whether with or without co-occurring mental illness or SUD, a comprehensive evaluation is required. This includes a detailed history taken by physicians, obtaining collateral information from family and friends, conducting physical examinations, and reviewing relevant laboratory tests [13].

ADHD and stimulant medications

Treatment for adult ADHD typically involves medication, behavioral interventions, skill development, and psychological counseling [14]. While medications can alleviate ADHD symptoms, they do not cure the condition. Stimulants are the most commonly used medications as they sustain neurotransmitter levels in the brain [15]. Non-stimulant medications, such as bupropion and atomoxetine, are also effective for treating ADHD in adults [16]. However, these non-stimulants tend to act more slowly than stimulants [17]. In our study, approximately 80% of patients with ADHD received stimulant medications. Other research indicates that these medications are effective in about 70-80% of individuals with ADHD [17]. Thus, stimulants remain a cornerstone of ADHD treatment, providing significant benefits in reducing symptoms and improving functional outcomes in both children and adults [18]. Nonetheless, their use necessitates careful consideration of efficacy, safety, and long-term outcomes [19]. The use of stimulant medications for ADHD has sparked debates regarding overdiagnosis, inappropriate prescribing practices, and potential misuse among individuals without ADHD [20]. Non-stimulant medications offer valuable alternatives for those who cannot use stimulants due to health issues or due to having adverse effects [17].

ADHD and the COVID-19 pandemic

Adults with ADHD experience higher levels of perceived or subjective stress compared to those without ADHD [21]. During the COVID-19 pandemic, adults with ADHD were disproportionately affected by stress related to lockdowns, compared to their non-ADHD counterparts [21,22]. The pandemic also impacted new patient assessments, delayed treatment initiation, reduced face-to-face appointments, and hindered physical monitoring of ADHD patients. Furthermore, many families faced economic hardships and increased family chaos, including the death of close family members [23]. Although it was anticipated that the prevalence of mental illness, including ADHD, would persist post-pandemic, few studies have addressed this issue. Our study found that ADHD cases remained consistently high even three years after the initial COVID-19 pandemic spike.

ADHD and gender bias

Females with ADHD are diagnosed less frequently than males, possibly due to a predominance of inattentive symptoms rather than hyperactivity ones [24]. Nevertheless, females with ADHD experience greater social and emotional difficulties resulting in poor self-esteem, more anxiety, and depression even from an early age [25,26]. Our study observed significantly higher numbers of ADHD cases among females compared to males. Clinical studies of children typically show a male-to-female diagnosis ratio of approximately 4:1, while community or population studies suggest a ratio closer to 2:1 [27]. There is growing evidence that part of the observed gender bias in diagnosis may be due to the under-recognition of ADHD in females, including delayed or missed diagnoses [28].

Limitations of the study

This retrospective study has limitations, including a restricted number of variables. Unlike prospective studies, retrospective studies cannot establish cause-and-effect relationships. Additionally, prospective studies are needed to further explore ADHD prevalence and characteristics in the post-COVID-19 era. The findings of this study may not be generalizable, as the sample consists of patients attending an urban outpatient mental health clinic. Further research involving diverse populations is necessary to gain a more comprehensive understanding of adult ADHD issues.

## Conclusions

The study demonstrates a sustained high number of ADHD diagnoses during and after the COVID-19 pandemic spike in 2020. Females exhibited a higher prevalence of ADHD, though stimulant medication use was consistent across genders. No significant age differences were observed between genders. It is beyond the scope of this study to determine whether the observed gender differences among ADHD-positive patients are due to increased awareness or actual biological predisposition. Future studies are needed to address these issues, as identifying gender differences will help allocate resources more effectively toward the target population.

## References

[1] American Psychiatric Association (2013). The Diagnostic and Statistical Manual of Mental Disorders, Fifth Edition. https://psycnet.apa.org/record/2013-14907-000.

[2] Leffa DT, Caye A, Rohde LA (2022). ADHD in children and adults: diagnosis and prognosis. Curr Top Behav Neurosci.

[3] Visser SN, Danielson ML, Bitsko RH (2014). Trends in the parent-report of health care provider-diagnosed and medicated attention-deficit/hyperactivity disorder: United States, 2003-2011. J Am Acad Child Adolesc Psychiatry.

[4] Spencer TJ, Biederman J, Mick E (2007). Attention-deficit/hyperactivity disorder: diagnosis, lifespan, comorbidities, and neurobiology. J Pediatr Psychol.

[5] Rogers MA, MacLean J (2023). ADHD symptoms increased during the COVID-19 pandemic: a meta-analysis. J Atten Disord.

[6] Breaux R, Dvorsky MR, Marsh NP (2021). Prospective impact of COVID-19 on mental health functioning in adolescents with and without ADHD: protective role of emotion regulation abilities. J Child Psychol Psychiatry.

[7] Reale L, Bartoli B, Cartabia M (2017). Comorbidity prevalence and treatment outcome in children and adolescents with ADHD. Eur Child Adolesc Psychiatry.

[8] Johnson J, Morris S, George S (2020). Attention deficit hyperactivity disorder in adults: what the non-specialist needs to know. Br J Hosp Med (Lond).

[9] Weiss M, Murray C (2003). Assessment and management of attention-deficit hyperactivity disorder in adults. Can Med Assoc J.

[10] Hansson Halleröd SL, Anckarsäter H, Råstam M, Hansson Scherman M (2015). Experienced consequences of being diagnosed with ADHD as an adult - a qualitative study. BMC Psychiatry.

[11] Katzman MA, Bilkey TS, Chokka PR, Fallu A, Klassen LJ (2017). Adult ADHD and comorbid disorders: clinical implications of a dimensional approach. BMC Psychiatry.

[12] (2024). Key substance use and mental health indicators in the United States: results from the 2019 National Survey on Drug Use and Health. https://www.samhsa.gov/data/sites/default/files/reports/rpt29393/2019NSDUHFFRPDFWHTML/2019NSDUHFFR090120.htm.

[13] Ruiz P, Strain EC, Langrod JG (2014). The Substance Abuse Handbook. https://clerkship.lwwhealthlibrary.com/book.aspx?bookid=1238&rotationId=0.

[14] (2024). Attention deficit hyperactivity disorder (ADHD) - diagnosis. https://www.nhs.uk/conditions/attention-deficit-hyperactivity-disorder-adhd/diagnosis/.

[15] (2024). Adult attention-deficit/hyperactivity disorder (ADHD) - diagnosis and treatment. https://www.mayoclinic.org/diseases-conditions/adult-adhd/diagnosis-treatment/drc-20350883.

[16] Sleath B, Sulzer SH, Carpenter DM (2014). Communication about ADHD and its treatment during pediatric asthma visits. Community Ment Health J.

[17] Kooij SJ, Bejerot S, Blackwell A (2010). European consensus statement on diagnosis and treatment of adult ADHD: the European Network Adult ADHD. BMC Psychiatry.

[18] Cortese S, Adamo N, Del Giovane C (2018). Comparative efficacy and tolerability of medications for attention-deficit hyperactivity disorder in children, adolescents, and adults: a systematic review and network meta-analysis. Lancet Psychiatry.

[19] Swanson J, Baler RD, Volkow ND (2011). Understanding the effects of stimulant medications on cognition in individuals with attention-deficit hyperactivity disorder: a decade of progress. Neuropsychopharmacology.

[20] Swanson JM (2018). Risk of bias and quality of evidence for treatment of ADHD stimulant medication. Clin Pharmacol Ther.

[21] Adamou M, Fullen T, Galab N, Mackintosh I, Abbott K, Lowe D, Smith C (2020). Psychological effects of the COVID-19 imposed lockdown on adults with attention deficit/hyperactivity disorder: cross-sectional survey study. JMIR Form Res.

[22] Lackschewitz H, Hüther G, Kröner-Herwig B (2008). Physiological and psychological stress responses in adults with attention-deficit/hyperactivity disorder (ADHD). Psychoneuroendocrinology.

[23] Horesh D, Brown AD (2020). Traumatic stress in the age of COVID-19: a call to close critical gaps and adapt to new realities. Psychol Trauma.

[24] Lau TW, Lim CG, Acharryya S, Lim-Ashworth N, Tan YR, Fung SS (2021). Gender differences in externalizing and internalizing problems in Singaporean children and adolescents with attention-deficit/hyperactivity disorder. Child Adolesc Psychiatry Ment Health.

[25] Johnston C, Mash EJ (2001). Families of children with attention-deficit/hyperactivity disorder: review and recommendations for future research. Clin Child Fam Psychol Rev.

[26] Santos S, Ferreira H, Martins J, Gonçalves J, Castelo-Branco M (2022). Male sex bias in early and late onset neurodevelopmental disorders: shared aspects and differences in autism spectrum disorder, attention deficit/hyperactivity disorder, and schizophrenia. Neurosci Biobehav Rev.

[27] Martin J (2024). Why are females less likely to be diagnosed with ADHD in childhood than males?. Lancet Psychiatry.

[28] Quinn PO, Madhoo M (2014). A review of attention-deficit/hyperactivity disorder in women and girls: uncovering this hidden diagnosis. Prim Care Companion CNS Disord.

